# Hybrid System of an Optical Nanofibre and a Single Quantum Dot Operated at Cryogenic Temperatures

**DOI:** 10.1038/s41598-018-31888-3

**Published:** 2018-09-10

**Authors:** K. Muhammed Shafi, Wei Luo, Ramachandrarao Yalla, Kazunori Iida, Emi Tsutsumi, Akiharu Miyanaga, Kohzo Hakuta

**Affiliations:** 1 Center for Photonic Innovations, University of Electro-Communications, Chofu, Tokyo 182-8585 Japan; 2 NS Materials Inc., Tsukushino, Fukuoka, 818-0042 Japan

## Abstract

Recent progress in quantum nanophotonics brings novel ways for manipulating single photons in various nano-waveguides. Among them, one promising approach is to use optical nanofibres (ONFs), tapered optical fibres with sub-wavelength diameter waists. Here, we develop a hybrid system of an ONF and a single quantum dot (QD) operated at cryogenic temperatures. We deposit a single colloidal CdSe QD on an ONF waist and observe emitted photons through the fibre guided modes. We systematically investigate emission characteristics for both the neutral exciton and charged exciton (trion) for one specific QD. We quantitatively show that the trion at cryogenic temperatures acts as an excellent quantum emitter for the ONF and QD hybrid system. The present ONF/QD hybrid system at cryogenic temperatures paves the way for quantum information technologies for manipulating single photons in fibre networks.

## Introduction

In recent years, various quantum nanophotonic systems have been developed in the context of single photon manipulation. A key point of such systems is to confine the light field modes to a tiny area to get a significant overlap between the light field and photoabsorption cross-section of a quantum emitter. Examples include photonic crystal waveguides and cavities^1–3^, whispering gallery mode cavities^4,5^ and plasmonic nanostructures^6,7^. In this context, one promising approach is to use optical nanofibres (ONFs), tapered optical fibres with sub-wavelength waist diameter^8^. In ONFs, nanofibre guided modes at the waist are strongly confined to a region with sub-wavelength dimensions. From a technical viewpoint, the ONF method has an advantage from other methods. Once a function has been implemented on a nanofibre, the function can be automatically integrated into fibre networks, since nanofibre guided modes evolve adiabatically to conventional fibre guided modes. Consequently, motivated by both physical and technical advantages, various hybrid systems of ONFs and quantum emitters have been developed for atoms^9^, molecules^10^ and solid state emitters^11–13^. Using the hybrid systems, various novel quantum optical processes have been demonstrated so far, such as efficient photon channeling of spontaneous emission into fibre guided modes^14,15^, high optical density with small number of atoms^16,17^ and atom trapping around a nanofibre^18,19^. Recently, such hybrid systems have been extended to nanofibre cavity quantum electrodynamics^20–22^ which may play a key role in the manipulation of single photons in fibre networks.

Regarding the emitter for quantum photonics, it must be a solid state emitter from a viewpoint of integrated photonic applications. In this view, one promising candidate would be colloidal semiconductor quantum dots (QDs), nanocrystals with core/shell structures in colloidal solutions, which can realize wide tunability of emission wavelength with a high quantum efficiency (QE). However, weak points for applying such QDs to photonic applications are a broad emission spectral width of the order of 10 nm and a rather long radiative decay time for the exciton transition^23–26^. Recently, however, it has been reported for CdSe QDs at an emission wavelength around 640 nm that both of these weak points can be removed by using thick shell structures for the core/shell QDs and extending the working temperature to cryogenic temperatures^27,28^. Spectral narrowing at cryogenic temperatures could obviously be expected, but a crucial point is that the main constituent for such QDs at cryogenic temperatures become charged nanocrystals^27,29^, which show a trion transition revealing one order shorter radiative decay time than that of the exciton transition for neutral nanocrystals^28,30^. So far, various investigations have been performed for such QDs. However, these previous investigations have been performed for QDs cast on a glass plate using microscope observation. Such measurement conditions are far from the real situation for photonic applications.

One may wonder about the cryogenic condition from the viewpoint of applications. However, recent progress in quantum photonics has proven that excellent properties which cannot be realized at room temperature can be realized at cryogenic temperatures using hybrid nanophotonic systems^1,2^. Moreover, in quantum information technologies, cryogenic technologies are becoming a standard method through superconducting single photon detectors^31^. In this view, it would be valuable to explore novel quantum photonic systems by extending the working condition to cryogenic temperatures.

In the present paper, we develop a hybrid system of an ONF and a single thick shell colloidal CdSe QD, which is operated at cryogenic temperatures. This system situation can be readily extended to photonic applications in fibre networks. By observing emitted photons through the fibre guided modes, we systematically investigate emission characteristics for exciton and trion transitions for one specific QD deposited on a nanofibre. We quantitatively confirm that the trion QD at cryogenic temperatures acts as an excellent quantum emitter for the ONF and QD hybrid system. The hybrid system of an ONF and a single thick shell CdSe QD at cryogenic temperatures paves the way for quantum information technologies for manipulating single photons in fibre networks.

## Results and Discussion

### Hybrid system at cryogenic temperatures

ONFs were fabricated by adiabatically tapering commercial single mode optical fibres using a heat and pull technique^32^. The nanofibre waist diameter was set to 320 nm so that emitted photons could be efficiently channeled into the fibre guided modes with an efficiency around 20%^15^. The nanofibre waist diameter was kept uniform for a length of 2.5 mm. Optical transmission of ONFs was >99%. We deposited a single QD on the nanofibre waist at three positions separated by 150 *μ*m. The deposition was done using a subpico-litre-needle-dispenser with a QD solution in toluene. The optical transmission of ONF did not show any observable change by the deposition. Regarding QDs, we synthesized thick shell QDs in toluene colloidal solution. The emission QE for the QDs was measured to be 85 ± 5% in colloidal solution using an integrating-sphere photometer^33^ (Otsuka Electronics, QE1100).

**Figure 1:**
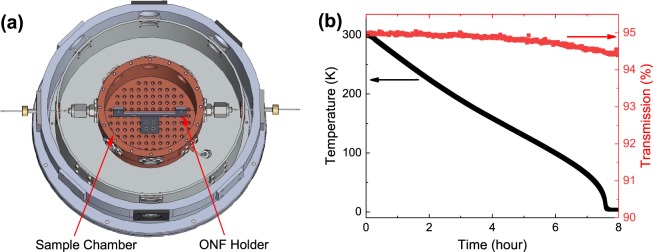
Hybrid system of ONF and QD at cryogenic temperatures. (**a**) Cross-sectional view of the cryostat. (**b**) Cooling characteristics versus time for the cryostat (black line) and for the optical transmission of an ONF (red line).

Regarding the cryogenic system, we used a custom designed optical cryostat, a cross-sectional view of which is shown in Fig. 1(a). The cryostat was cooled by a pulse-tube refrigerator. The ONF with single QDs was installed in a sample chamber made of copper, which was fixed to a cold plate at the bottom of the cryostat. The ONF was mounted on a U-shaped holder with an opening length of 70 mm to keep the ONF straight. The ONF was fixed to the holder by mechanically sandwiching it with a small piece of silica plate at the top edge of each U-pillar and the holder was fixed to the sample chamber. In order to keep the ONF immune to the thermal contraction due to the cooling, the U-shaped holder was made of silica. The QDs on a nanofibre were cooled down through the buffer-gas cooling by filling the sample chamber with He-gas at a pressure of 15 kPa at room temperature. By installing the ONF into the cryostat, the optical transmission of the ONF decreased to 95%, essentially due to the mechanical sandwiching of the ONF to the U-pillars.

Figure 1(b) shows the cooling characteristics of the cryostat (black curve) and the optical transmission of the installed ONF (red curve) versus temperature. The temperature was measured at the inside bottom of the sample chamber. The transmission was measured at a wavelength of 640 nm. By starting the cooling, the temperature was lowered gradually and reached the lowest attainable temperature of the cryostat, 3.7 K, after about 8 hours. Since the cooling process was slow, it was reasonably assumed that the sample chamber, He-gas, ONF and QDs were all in thermal equilibrium and the QD temperature was assumed to be the same as the temperature of the sample chamber. We observed that the optical transmission was unchanged at the room temperature value for temperatures down to 150 K. For further cooling, although the transmission showed a gradual decrease, the drop was very small (0.5% at 3.7 K), which might be negligible for photonic applications.

### Emission characteristics of a single QD on a nanofibre

**Figure 2:**
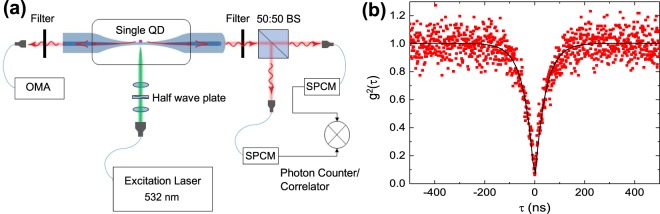
Hybrid system of an ONF and a single QD. (**a**) Measurement setup for the hybrid system. (**b**) Photon correlation characteristics for a single QD in the hybrid system.

Figure 2(a) illustrates a measurement scheme for the emission characteristics of the QDs on a nanofibre. The QDs were excited perpendicularly to the nanofibre with a cw or pulsed frequency-doubled YAG laser at a wavelength of 532 nm. The cw laser was a single-frequency laser and the pulsed laser was a mode-locked laser with a pulse width of 10 ps. The laser beam was focused to the nanofibre with a spot diameter of 15 *μ*m and the focus spot position was adjusted to one of the three QDs separated by 150 *μ*m on the nanofibre by scanning the laser beam along the nanofibre. The laser was linearly polarized with a polarization perpendicular to the nanofibre axis. Emitted light was measured through the nanofibre after passing through a 560 nm long pass filter (O56, HOYA) to remove the scattered laser light. One side of the ONF was introduced to an optical multichannel analyzer (OMA) spectrometer with a focal length of 35 cm for the spectral measurements. We used two gratings of 300 and 1,800 lines/mm, leading to instrumental widths of 0.83 ± 0.03 and 0.11 ± 0.02 nm, respectively. The instrumental widths were obtained by measuring the spectral width of a He-Ne laser light at 633 nm which has a sufficiently narrow spectral width of 2 pm. The other side of ONF was used for photon counting/correlation measurements with two single-photon counting modules (SPCMs). The photon arrival times at the two SPCMs were recorded using a time tagging module (Pico-Harp 300, Pico Quant GmbH). By analyzing the arrival times, we obtained photon counting-rates and photon correlations. The temporal behaviour of the QD emission was measured by excitation with the pulse laser. The emission decay behaviour was obtained by measuring correlations between the exciting laser pulse and emitted photons. The temporal resolution of the system was 290 ps.

We measured the photon correlation function *g*^(2)^(*τ*) for the three QD depositions on the nanofibre, where *τ* is the photon arrival time difference at the two SPCMs. A measured result is displayed in Fig. 2(b). One can readily recognize a sharp dip at zero time-difference with *g*^(2)^(0) = 0.05 ± 0.01, revealing that the emitter is a single quantum emitter. For the other two depositions, similar behaviours with a sharp central dip of *g*^(2)^(0) = 0.06 ± 0.01 and 0.08 ± 0.01 were observed. All the following emission characteristics were obtained for one specific single QD which gave the photon correlation displayed in Fig. 2(b).

**Figure 3:**
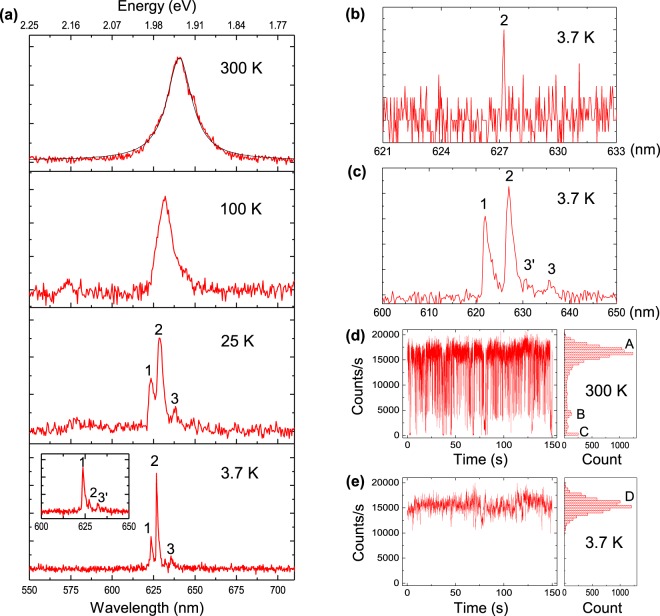
Emission characteristics for a single QD on a nanofibre in the hybrid system. All data shown here were obtained with a cw laser excitation. (**a**) Temperature dependence of the QD measured with an integration time of 30 s. Peak 1 and 2 correspond to ZPLs for exciton and trion, respectively. Peak 3 and 3′ are LO-phonon replica of trion and exciton, respectively. (**b**) Emission spectrum measured at 3.7 K with an integration time of 1 s. (**c**) Emission spectrum measured at 3.7 K with an integration time of 270 s. (**d**) Photon counting behaviour measured at room temperature for a period of 150 s. Three peaks (A, B, C) in the histogram correspond to exciton, trion and dark count, respectively. (**e**) Photon counting behaviour measured at 3.7 K for a period of 150 s. The histogram shows a single peak marked by D.

Figure 3(a) shows the temperature dependence for the QD emission spectrum from room temperature to 3.7 K. All spectra were measured with an integration time of 30 s using a grating of 300 lines/mm at an excitation intensity of 30 W/cm^2^. At room temperature, the spectrum shows a symmetric profile at a central wavelength of 640.4 nm with a width of 19.4 ± 0.2 nm FWHM. By lowering the temperature to 100 K, the spectral shape becomes asymmetric with a narrower FWHM and shows a blue shifting of the peak wavelength. At around 25 K, the spectrum starts to split into three peaks as marked by 1, 2 and 3 and three well resolved peaks are observed with FWHMs of 1.2 nm at 3.7 K. The observed energy separation between peak 1 and 2 was 15.2 meV (5.0 nm in wavelength), leading to the assignment of the two peaks as zero phonon lines (ZPLs) for the exciton of a neutral CdSe nanocrystal and for the trion of a charged CdSe nanocrystal, respectively^30^. Regarding peak 3, it was red-shifted by 26.5 meV from peak 2 and was attributed to a longitudinal optical phonon (LO-phonon) replica of the trion ZPL^34^. Since the measurements were performed for one QD, the observed peak intensity ratio directly reflects the occurrence probability of the neutral and charged conditions for the observation period of 30 s at a specific timing. The inset shows another example of the spectrum observed at a different timing. For this case, the neutral condition is much more probable than the charged condition. Note that peak 3′ is the LO-phonon replica of the exciton ZPL, peak 1.

In order to examine the spectral width, the emission spectrum at 3.7 K was measured for shorter integration times with a grating of 1,800 lines/mm. Figure 3(b) shows the spectrum observed with an integration time of 1 s. Although the S/N-ratio is not good due to a low dispersion efficiency of the 1800 lines/mm grating, one can readily recognize a sharp peak at the trion ZPL wavelength. The observed width was 0.12 ± 0.02 nm FWHM (~350 *μ* eV FWHM), close to the instrumental width of the OMA spectrometer. Since spectral profiles measured through a spectrometer can be expressed by a convolution of the instrumental and emission spectral profile, the resulting spectral width can be expressed approximately as a sum of the instrumental and emission spectral widths. Thus, we expect that the emission spectral width for the 1 s integration time is much narrower than the measured value. Such spectral narrowing for shorter integration times is understood as due to the spectral diffusion and the present observations are consistent with previous results obtained for similar QDs^34,35^.

The spectrum at 3.7 K was also measured for longer integration times with a grating of 300 lines/mm. The observed spectral width showed a saturation behaviour, reaching a constant value for longer integration times than 100 s. Figure 3(c) shows a spectrum measured for an integration time of 270 s. One can see exciton and trion ZPLs with corresponding LO-phonon replica. Spectral widths for both the exciton and the trion were 1.6 ± 0.1 nm FWHM and were 30% broader than those observed with an integration time of 30 s. This width corresponds to the saturated width for the spectral diffusion. Note that the intrinsic saturation width for the spectral diffusion may be estimated as 0.8 nm FWHM, since the measured width can be approximated as a sum of the instrumental and the emission spectral widths and the instrumental width for a grating of 300 lines/mm is 0.83 ± 0.03 nm. Regarding the peak intensity ratio between the trion and the exciton, one can readily see that it changes according to the integration time. Only one peak (exciton or trion) was observed for an integration time of 1 s and for 30 s integration time, although both the exciton and the trion were observed, the ratio drastically changed by the observation timing. By increasing the integration time longer than 100 s, the ratio reached a constant value which does not depend on the observation timing. This means that the occurrence of the exciton and the trion reaches a stationary condition for an integration time longer than 100 s. We estimated the occurrence probability for the exciton and the trion at 3.7 K to be 41 ± 3 and 59 ± 3%, respectively, from the peak ratio observed at 270 s integration time in Fig. 3(c) for the present QD.

Figure 3(d,e) display photon counting behaviours measured for a period of 150 s at room temperature and at cryogenic temperature of 3.7 K, respectively. A histogram for the count rate is shown at the right side of each figure. At room temperature, one can clearly see three steps in the count rate and the three steps are marked by A, B and C in the histogram. The highest count rate, peak A of the histogram was 16,500 ± 1,100 counts/s. Count rate of peak B was 4,400 ± 450 counts/s, which was 26 ± 3% of the peak A. Peak C was 250 ± 60 counts/s, which was the dark count rate of SPCM and corresponds to the so-called blinking. The occurrence probability for peak A, B and C which was obtained by measuring the area of each peak was 89 ± 6, 8 ± 0.5 and 3 ± 0.5%, respectively. This means that the occurrence probability of blinking was rather small, 3% at room temperature for the present QD. We attributed the peak A and B to the neutral exciton emission and the trion emission, respectively^36,37^. The peak count ratio for B to A is 0.26, leading to a fact that emission QE for the trion is 26 ± 3% of that for the exciton.

It would be meaningful to estimate the lower limit of the exciton QE for the QD picked-up onto the ONF. Since the QE value was measured for the liquid solution as 85 ± 5%, we assume that the lower limit of QE for a single QD is 80%. Note that the measured QE is an effective average value for a mixture of excitons and trions. This situation can be formulated as follows. The photon emission rate for the exciton *n*_*e*_ (trion *n*_*t*_) can be written under a stationary condition as *n*_*e*_ = *η*_*e*_*N*_*e*_ (*n*_*t*_ = *η*_*t*_*N*_*t*_) where *η*_*e*_(*η*_*t*_) and *N*_*e*_ (*N*_*t*_) are the QE and excitation rate for exciton (trion), respectively. The total photon emission rate *n* for the mixture can be written as *n* = *η*_*eff*_*N* = *η*_*eff*_ (*N*_*e*_ + *N*_*t*_) where *η*_*eff*_ and *N* are the effective QE for the mixture and the total excitation rate for the exciton and the trion, respectively. The total emission rate *n* can also be expressed as *n* = *n*_*e*_ + *n*_*t*_. By using these relations, one can relate the exciton QE to the effective QE as following.

1 $${\eta }_{e}=[{\alpha }^{-1}-({\alpha }^{-1}-1)\frac{{n}_{e}}{n}]{\eta }_{eff}$$

where *α* is a QE ratio between the trion and the exciton. It should be mentioned that *n*_*e*_/*n* corresponds to a stationary occurrence probability ratio between the exciton and a sum of the exciton and the trion. By using the experimentally obtained parameters; *α* = 26 ± 3%, *n*_*e*_/*n* = 92 ± 6% and the lower limit of *η*_*eff*_ = 80%, we estimated the lower limit of the exciton QE at room temperature to be 96% including errors in the measured values.

By lowering the temperature below 50 K, the step behaviour in the count rate disappeared; the count rate showed a simple one peak behaviour at cryogenic temperatures. An example measured at 3.7 K is displayed in Fig. 3(e). The count rate at the center was 15,500 ± 1,300 counts/s. It should be mentioned that, as described for the observation on Fig. 3(c), the stationary occurrence probabilities for the neutral and the charged condition at 3.7 K to be 41 and 59%, respectively. So, the one peak behaviour at cryogenic temperatures means that the QE of the charged trion increased by lowering the temperature and reached an almost equal value as that of the neutral exciton at cryogenic temperatures. Since the peak count rate at 3.7 K was 6% lower than that of the neutral exciton at room temperature, we estimated the QE value at cryogenic temperatures to be higher than 90%, since the exciton QE at room temperature was higher than 96%.

Next, we describe temporal behaviour of the emission measured by excitation with the 10-ps laser-pulse. The repetition rate of the laser pulse was 0.5 × 10^6^ pulses/s and the measurement time was 200 s for each decay measurement. First we describe the results for cryogenic temperatures from 3.7 to 40 K. Measurements were carried out using two types of interference filters. One filter was a narrow band filter with an FWHM of 2 nm which could resolve the exciton and trion peaks separated by 5.0 nm by adjusting the filter tilt-angle. The narrow band filter was used for the measurements at 3.7 K where the exciton and trion peaks were well resolved for a long measurement time, as shown in Fig. 3(c). The other filter which had an FWHM of 10 nm was used for measuring the temperature dependence of the decay profile. Note that emissions from the exciton and the trion were mixed together for the measurements with this filter.

**Figure 4:**
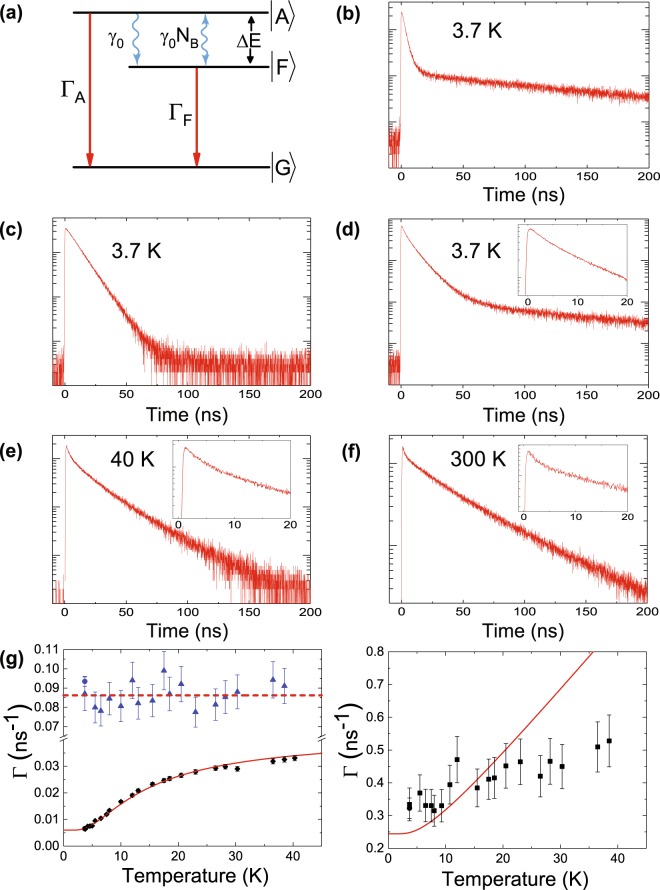
Temporal characteristics for a QD on a nanofibre. (**a**) Schematic digram for the exciton transition, the upper state of which consists of two levels referred as bright state |*A*> and dark state |*F*>. (**b**) Exciton decay profile measured at 3.7 K with an interference filter of 2 nm FWHM. (**c**) Trion decay profile measured at 3.7 K with an interference filter of 2 nm FWHM. (**d**,**e**) Decay profile measured at 3.7 K (40 K) with an interference filter of 10 nm FWHM. Emissions from both exciton and trion are mixed together. Inset shows an expanded view up to 20 ns. (**f**) Decay profile measured at 300 K with an interference filter of 40 nm FWHM at 650 nm. (**g**) Left Panel: Temperature dependence for the decay rate of trion (blue colour: triangles and a circle) and slow exciton (black colour: diamonds and a circle). Right Panel: Temperature dependence for the decay rate of fast exciton (black colour: squares and a circle). For both panels, decay rates marked by circles were obtained from the 2 nm FWHM filter measurements and others were from the 10 nm FWHM filter measurements.

It has been understood for CdSe core/shell QDs that the upper state of the exciton consists of two nearby levels referred to as bright and dark states which mix together via phonons at cryogenic temperatures^34,38^. On the other hand, the upper state of the trion is effectively one level at cryogenic temperatures^29,39^. In Fig. 4(a), the energy diagram of the exciton transition is schematically illustrated. Bright and dark states are specified as |*A*> and |*F*>, respectively, with an energy splitting of ΔE. Photon transitions are specified by red arrows with decay rates of Γ_*A*_ and Γ_*F*_. Phonon transitions are specified by blue wavy arrows. *γ*_0_ denotes the spontaneous phonon decay rate and the induced phonon transition rate is expressed as *γ*_0_ *N*_*B*_(*T*), where *N*_*B*_(*T*) denotes the Bose-Einstein phonon number at temperature *T*.

Figure 4(b,c) show the decay profiles for exciton and trion, respectively, measured at 3.7 K using the narrow band filter. The decay profile for exciton in Fig. 4(b) clearly shows a double exponential behaviour, with a fast decay time of 3.1 ± 0.3 ns and slow decay time of 153 ± 5 ns. The observed double exponential behaviour was well explained by the two level scheme shown in Fig. 4(a). On the other hand, the trion decay profile in Fig. 4(c) shows clearly a single exponential decay with a decay time of 10.7 ± 0.3 ns. The single exponential decay was consistent with a single upper state model of the trion.

Decay profiles at 3.7 and 40 K measured with the 10 nm FWHM filter are displayed in Fig. 4(d,e), respectively. In Fig. 4(d), one can briefly recognize two exponential decay curves corresponding to the decay processes for trion and slow exciton. However, the fast exciton decay curve was almost masked by the trion decay process. By expanding the behaviour in shorter time range as in the inset, the situation can be seen clearly. A similar situation was seen for the decay curve measured at 40 K in Fig. 4(e). We analyzed the decay curves obtained with the 10 nm FWHM filter by fitting with a sum of three exponential decay curves. We derived the trion decay time and the slow exciton decay time reliably, but we evaluated the reliability for the derived fast exciton decay time much lower than that for the other two decay times.

Obtained decay rates Γ(=1/*τ*) are plotted in Fig. 4(g) versus temperature from 3.7 to 40 K with error bars which were estimated from the fitting. In the left panel, the decay rates for the trion and the slow exciton are plotted by blue triangles (circle) and black diamonds (circle), respectively, measured with the 10 nm (2 nm) filter. The right panel plots the fast decay rate by black squares (circle) measured with the 10 nm (2 nm) filter. Error bars are ±3% for both the slow exciton decay rates and the trion decay rate measured with the 2 nm filter, ±10% for both the trion decay rates measured with the 10 nm filter and the fast exciton decay rate measured with the 2 nm filter and ±15% for the fast exciton decay rates measured with the 10 nm filter. Regarding the exciton decay, we analyzed the decay behaviours using the two state (bright and dark state) model in Fig. 4(a), which gives the following analytical expression for the decay rate^34^.

2 $${{\rm{\Gamma }}}_{\pm }=\frac{1}{2}\{{{\rm{\Gamma }}}_{A}+{{\rm{\Gamma }}}_{F}+{\gamma }_{0}\,\coth (\frac{{\rm{\Delta }}E}{2{k}_{B}T})\pm \sqrt{{({{\rm{\Gamma }}}_{A}-{{\rm{\Gamma }}}_{F}+{\gamma }_{0})}^{2}+{\gamma }_{0}^{2}{\sinh }^{-2}(\frac{{\rm{\Delta }}E}{2{k}_{B}T})}\}$$

where Γ_±_ are observable fast and slow decay rates for the exciton system, respectively. We fitted the measured slow and fast decay rates from 3.7 to 40 K simultaneously by the above expressions for Γ_−_ and Γ_+_, respectively and determined four parameters; Γ_*A*_, Γ_*F*_, ΔE and *γ*_0_, for the present QD using a nonlinear least-square fitting. We used the error bar of each data point as its weight. The obtained values with one standard deviation errors were Γ_*A*_ = 7.94 ± 0.27×10^−2^ ns^−1^, Γ_*F*_ = 6.01 ± 0.30×10^−3^ ns^−1^, ΔE = 1.38 ± 0.07 meV and *γ*_0_ = 0.165 ± 0.013 ns^−1^ and were in good correspondence to the values obtained for similar CdSe QDs^25,34^. The fitted curves for Γ_−_ and Γ_+_ were drawn by a red line in Fig. 4(g). The fitted curve for the slow exciton reproduced the observations quite well showing a gradual increase to reach a saturation. Regarding the fast decay rate, although a tendency to increase as the temperature increase was observed for both the measured and the fitted results, the discrepancy between them was rather large, especially in the temperature range higher than 20 K where faster decay rates are expected. This discrepancy can be understood by the low reliability of the measured decay rates for the fast exciton. On the other hand, the measured trion decay rates did not show any observable temperature dependence. This behaviour was naturally understood from a fact that the upper state of trion is a single state and is free from phonon interaction at cryogenic temperatures^29,39^. Note that the measured values showed about ±10% scatter around the average value which is much larger than the scatter for the plot for the slow exciton. Since the trion decay curve is perturbed by the fast and slow exciton decay curves in the falling and trailing region, respectively, the reliability for the fitted trion decay time should be lower than that for the slow exciton. This situation can be seen at 3.7 K where the trion decay was measured under both conditions of trion only and trion/exciton mixed. The two measured points show 8% deviation, which may give another measure for the reliability for the value measured under the mixed condition. We obtained the trion decay rate as a constant value of 0.086 ± 0.009 ns^−1^ (decay time of 11.6 ± 1.1 ns).

Regarding the upper state splitting of the exciton, it should be noted why we did not observe the splitting for the exciton emission spectrum in Fig. 3(a). This can be understood as being due to the instrumental width of the spectrometer; the splitting for the bright and dark state of 1.38 meV corresponds to a splitting of 0.4 nm in wavelength at 640 nm, which is not resolvable with the 300 lines/mm grating.

Lastly, in Fig. 4(f) we show a decay curve measured at 300 K using an interference filter with an FWHM of 40 nm at 650 nm. The decay profile shows a double exponential behaviour, although the fast component is rather small. We obtained the fast decay time as 3.0 ± 0.3 ns and slow decay time as 43.0 ± 0.6 ns. The slow decay time can be understood as the exciton decay time at room temperature. It should be noted that the room temperature decay time is not described by Eq. (2), since the two state model of the exciton is valid for the temperatures lower than 100 K^40,41^. The fast decay component can be understood as the trion decay time at room temperature. Note that the trion decay time is four times shorter than the value at cryogenic temperatures. This observation is reasonable. Since the QE of trion is reduced to 26% of the exciton at 300 K, the excited trion population mainly flows into nonradiative channels at 300 K and the trion decay time at 300 K should be about four times faster than that at cryogenic temperatures where the QE is higher than 90%.

## Conclusion

We have demonstrated that the hybrid system of an ONF and a thick shell QD works successfully at cryogenic temperatures. We have shown that the ONFs keep high optical transmission at cryogenic temperatures and that one single QD on the ONF waist reveals good emission characteristics with a high QE of 94% at cryogenic temperatures. Regarding the exciton or trion issue, the trion is superior to the exciton, since the trion shows a single decay process with a decay rate larger than the slow decay rate of the exciton. Regarding the occurrence probability, however, the trion probability is 59% for the present QD, but the probability may be improved close to 100% by introducing a photochemical electronic doping to the synthesis of QDs as reported in ref.^42^.

The hybrid system of an ONF and a single QD at cryogenic temperatures would be a promising candidate for manipulating single photons in fibre networks. Since solid state quantum emitters generally show much better characteristics at cryogenic temperatures, the present system can be extended to apply to various quantum emitters in various wavelength ranges. We anticipate that the hybrid system at cryogenic temperatures may open a new route for the realization of quantum networks.

## Methods

### Preparation of thick shell quantum dots (QDs)

QDs with a core-shell structure, which emit 640 nm fluorescence, were synthesized in a lab scale using a similar method in ref.^43^. Core structure, which is mainly composed of cadmium (Cd) and selenium (Se), was first synthesized by reacting a Cd based compound and Se at high temperature (above 250 °C) in a hydrophobic organic medium. The core-CdSe was separated by precipitation with ethanol and purified by washing two times, followed by centrifugation (7,500 rpm). Protection of the core-CdSe with inorganic shell, which is mainly composed of zinc (Zn) and sulphur (S), was performed by reacting Zn based compounds and S at high temperature above 280 °C. The reaction was repeated at least two times, in order for generating a thicker shell structure (more than 5 nm thickness around the core). The size and shape of the core-QD and the resulting core-shell structured QD were confirmed by the observation of scanning electron microscopy images. The core-shell structured QD was purified by a combination of precipitation and centrifugation (7,500 rpm), then stored in toluene as a uniform dispersion.

### Deposition method of a single quantum dot (QD)

Single QDs were deposited on a nanofibre using a sub-pico-litre needle dispenser installed on an inverted microscope^11^. An ONF was mounted on a U-shaped holder, which was fixed to a motor-controlled *x*-*y* stage on the microscope. A laser beam was introduced into the ONF for monitoring the deposition process. The dispenser consisted of a tapered glass-tube and a tungsten needle with a tip of 5 *μ*m diameter and the needle was set inside the glass tube. Internal volume of the glass tube was about 5 mm^3^ and was filled with a toluene solution of QDs (concentration of 10^13^ dots/cm^3^). The glass-tube end was set 200 *μ*m above the nanofibre. The needle was computer-controlled by monitoring scattered laser light from a deposited spot through the microscope. Once the needle tip passed through the glass tube, it carried a small amount of QD solution at its edge and the needle tip just touched the nanofibre surface to deposit a single QD. The needle speed was adjusted to 100 *μ*m/s so that the tip could touch the nanofibre surface before toluene on the tip dried up. Spatial accuracy for the deposition along the nanofibre axis was estimated to be 0.4 *μ*m and the success probability of one dot deposition for each trial was about 60%.

## Data Availability

Data are available on reasonable request.
